# Neoadjuvant Chemotherapy With Capecitabine Plus Cisplatin in Patients With Locally Advanced Nasopharyngeal Cancer: Case Series Study

**DOI:** 10.1200/JGO.2016.006924

**Published:** 2016-11-16

**Authors:** Reyad Dada, Mohamed El Sayed, Jamal Zekri

**Affiliations:** **Reyad Dada**, **Mohamed El Sayed**, and **Jamal Zekri**, King Faisal Specialist Hospital and Research Center, Jeddah; **Reyad Dada** and **Jamal Zekri**, Al-Faisal University, Riyadh, Kingdom of Saudi Arabia; and **Mohamed El Sayed**, Cairo University, Cairo, Egypt.

## Abstract

**Purpose:**

Capecitabine, an oral fluorouracil (5-FU) derivative, has replaced 5-FU in many chemotherapy regimens used in various GI tract cancers. The experience with capecitabine in nasopharyngeal carcinoma (NPC) is limited.

**Patients and Methods:**

We report on eight patients with locally advanced NPC treated with neoadjuvant chemotherapy with capecitabine and cisplatin.

**Results:**

All eight patients responded well to the chemotherapy combination and achieved complete remission after definitive chemoradiotherapy. No grade 3/4 toxicities were observed. Five patients experienced a relapse after 6, 8, 9, 12, and 17 months.

**Conclusion:**

In the patients studied, capecitabine (in combination with cisplatin) was a safe and effective substitution for 5-FU for the neoadjuvant treatment of locally advanced NPC. Larger prospective clinical studies are required to confirm these results.

## INTRODUCTION

Nasopharyngeal carcinoma (NPC) is a chemotherapy-sensitive cancer and more common in Asia, with a reported annual incidence in Far Asia of approximately 25-fold higher than in the Western world.^1^ Epstein-Barr virus plays an important role in the pathogenesis of NPC and induces tumor metastasis.^2^ Concurrent chemoradiotherapy (CCRT) is the standard for locally advanced NPC (LANPC). However, neoadjuvant chemotherapy (NAC) with cisplatin and 96 hours of infusional fluorouracil (5-FU) may reduce the radiotherapy field, and thus toxicity, and may improve long-term outcomes in select high-risk patients.^3^

Conventional infused 5-FU is an essential part of many chemotherapy regimens used to treat patients with head and neck cancer. At many institutions, it requires central venous access and hospitalization or the use of an ambulatory portable chemotherapy pumps (APCP), which causes substantial inconvenience to patients. Furthermore, central venous catheters can cause immediate and long-term complications, including pneumothorax, thrombosis, and sepsis.^4^

Capecitabine is an oral fluoropyrimidine carbamate that is metabolized to 5-FU through three steps of enzymatic reactions. It has replaced 5-FU in many chemotherapy regimens used to treat patients with various GI tract cancers. Several trials have demonstrated in patients with metastatic colorectal cancer that adjuvant capecitabine is well tolerated and has much of the same antitumor activity as 5-FU.^5-7^ Capecitabine is incorporated in chemotherapy regimens for many other tumor types, such as esophageal,^8^ gastric,^9^ and breast.^10^ Single-agent and combination regimens have also shown advantage in other cancer types, such as prostate, pancreatic, renal cell, and ovarian.^11^ The efficacy, safety, and convenience of the oral formulation make capecitabine an attractive option for patients with NPC.

## PATIENTS AND METHODS

Cisplatin and 96 hours of infusional 5-FU is the standard regimen for the neoadjuvant treatment of patients with LANPC at our hospital. The treatment is administered intravenously in an inpatient setting or through an outpatient APCP. Intravenous 5-FU is substituted with oral capecitabine twice per day on days 1 to 14 every 3 weeks for patients who refuse hospital admission, the placement of a central venous catheter, and the APCP. Patients who achieve excellent clinical response after the second cycle undergo early radiologic examination to confirm their response and are referred for radical CCRT and not undergoing the third cycle of NAC. Thereafter, all patients receive radical combined-modality chemotherapy (cisplatin 100 mg/m^2^ once every 3 weeks) and radiation with the intensity-modulated radiation therapy administered according to standard hospital practice guidelines.

The follow-up protocol includes clinical examination with endoscopy every 2 to 3 months during the first year, every 4 to 6 months the second year, and every 6 to 12 months thereafter. Radiologic examination starts 2 to 3 months after the end of radiotherapy and is repeated every 6 months for the first 2 years.

We performed a retrospective case series study to analyze the outcome of patients with LANPC treated with neoadjuvant capecitabine and cisplatin followed by definitive CCRT.

## RESULTS

Between March 2013 and June 2016, neoadjuvant capecitabine (with cisplatin) was administered to eight patients (six male and two female) with LANPC. Mean age was 47.8 years. All patients completed the neoadjuvant and combined-modality treatment. As a result of excellent clinical response in four patients, the induction therapy was stopped after two cycles. Computed tomography scans showed 25% complete and 85% partial remissions after NAC. Treatment was well tolerated without grade 3 to 4 toxicities. Furthermore, no dose reduction or delay of NAC cycles was necessary. No toxicity-related hospitalization occurred during NAC. All patients started radical combined-modality chemoradiotherapy (intensity-modulated radiation therapy) within 4 to 6 weeks after NAC. Radiologic response assessment was carried out 2 to 3 months after completion of the therapy. All patients achieved clinical and radiologic complete remission by the end of the planned treatment approach (NAC and CCRT). At the last follow-up (August 2016), three patients remained disease free and five experienced a relapse 6, 8, 9, 12, and 17 months after completing CCRT. All patients were alive at the last follow-up. Table 1 lists the patient characteristics and relevant adverse effects.

**Table 1:**
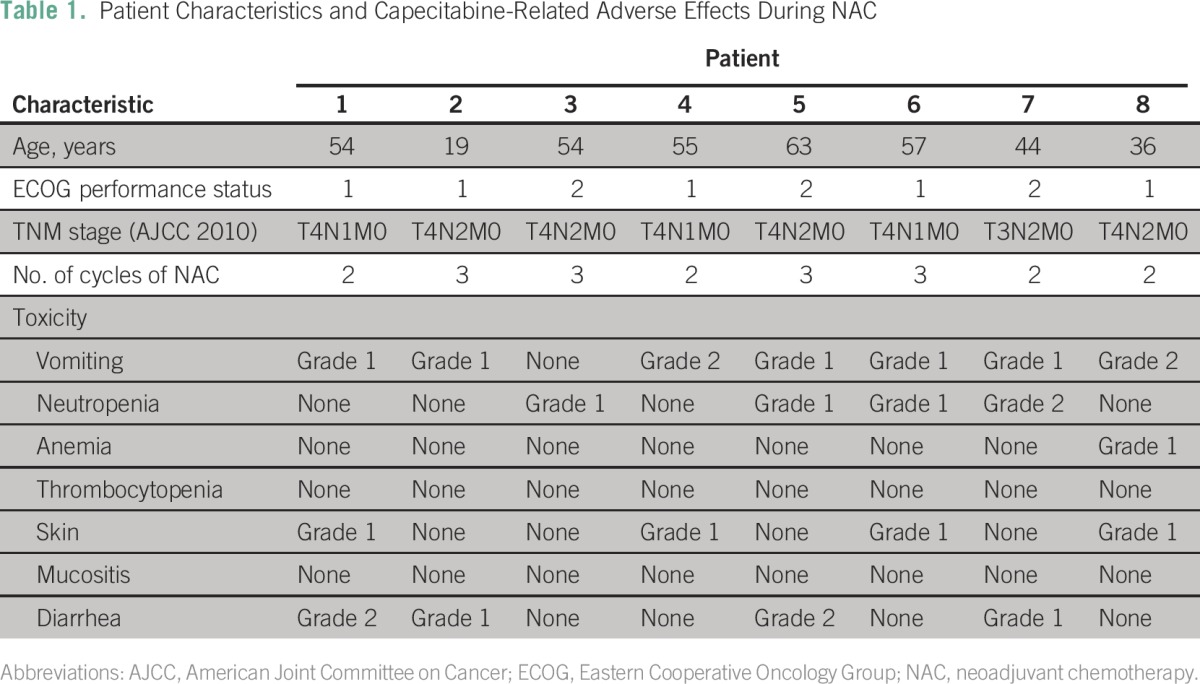
Patient Characteristics and Capecitabine-Related Adverse Effects During NAC

## DISCUSSION

The patients treated with capecitabine had an excellent outcome with an acceptable adverse effect profile. There was no grade 3 to 4 toxicity. Although the drug is used in many GI tract chemotherapy protocols instead of 5-FU, it has not yet replaced 5-FU in chemotherapy regimens used in patients with head and neck cancer. Several phase II studies confirmed the effectiveness and tolerance of capecitabine combination in head and neck cancer.^12-16^ The recommended dose range of capecitabine in these studies was 825 to 1,250 mg/m^2^. In addition, in the concurrent setting with radiotherapy, capecitabine seems to be effective with a manageable adverse effects profile.^17,18,19^ A randomized study in 153 patients with locally advanced squamous cell head and neck cancer showed a significantly better rate of complete response and better overall response with concurrent cisplatin and capecitabine plus radiotherapy compared with cisplatin and 5-FU plus radiotherapy, with similar progression-free and overall survival.^20^

Data on capecitabine in NPC are scarce and mostly limited to patients with metastatic and refractory disease. These data from four small phase II studies examined the outcome and tolerance to capecitabine in metastatic/refractory NPC. The efficacy results were similar to conventional 5-FU but with better patient acceptance and, in general, a similar tolerability profile.^21-24^

To our knowledge, no data in the literature describe the use of capecitabine in neoadjuvant treatment of NPC apart from preliminary results of one recently reported study from China. In this randomized study in patients with LANPC, capecitabine and cisplatin were better tolerated than 5-FU and cisplatin (neutropenia and electrolyte disturbance) and were associated with better overall survival (hazard ratio, 0.57; 95% CI, 0.34 to 0.97).^25^

Some patients with head and neck cancer can present with dysphagia, which may preclude the routine use of oral formulation drugs. In patients without dysphagia, capecitabine oral treatment is considered an attractive and practical substitute to a long course of intravenous 5-FU infusion. The patients in this study expressed satisfaction with this treatment because it was well tolerated and allowed them more time away from the hospital. This approach relieves the burden on already-stretched health care resources by reducing bed occupancy. Furthermore, the treatment is more convenient and safe for patients because it avoids the need for a central line that may be associated with complications.

In conclusion, capecitabine is an active and safe substitute for 5-FU in patients with LANPC treated in a neoadjuvant setting. Further validation in randomized clinical studies in patients with LANPC and metastatic NPC is required.
